# First person – Tylor Lewis

**DOI:** 10.1242/dmm.049984

**Published:** 2022-12-01

## Abstract

First Person is a series of interviews with the first authors of a selection of papers published in Disease Models & Mechanisms, helping researchers promote themselves alongside their papers. Tylor Lewis is first author on ‘ Microvesicle release from inner segments of healthy photoreceptors is a conserved phenomenon in mammalian species’, published in DMM. Tylor is a postdoc in the lab of Vadim Arshavsky at Duke University Medical Center, Durham, NC, USA, investigating the biology of the visual system and the pathophysiological mechanisms of retinal disease.


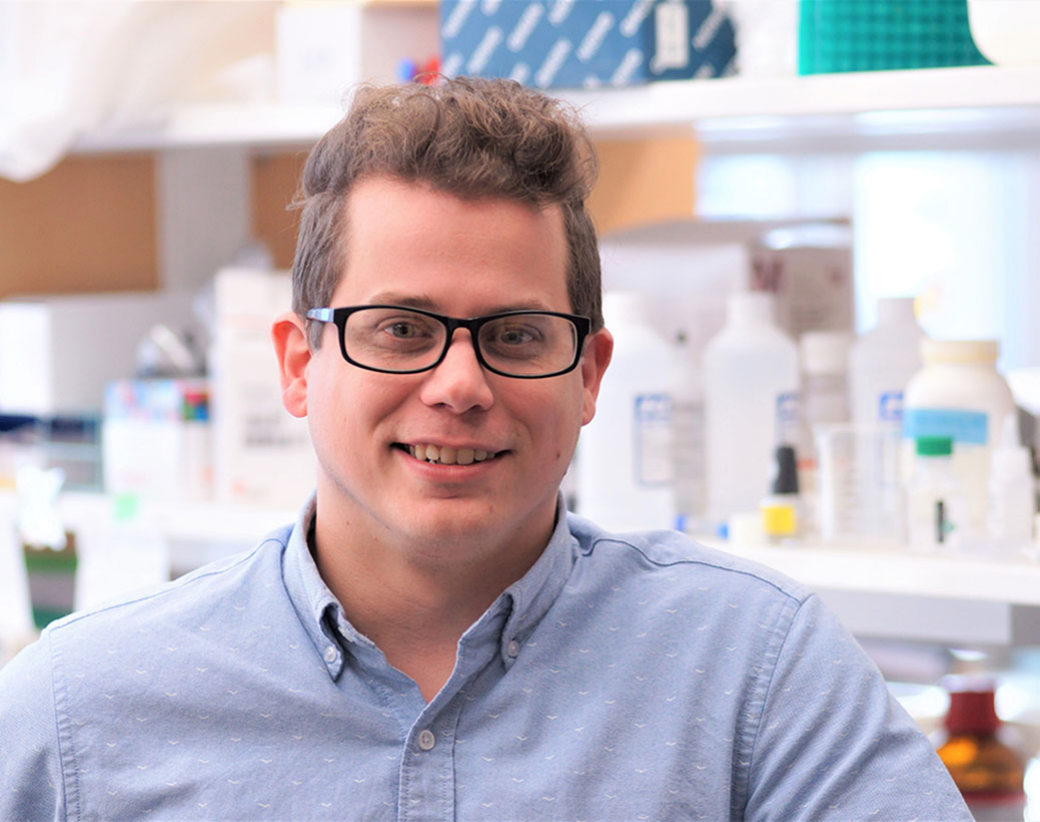


**Tylor Lewis**

**How would you explain the main findings of your paper to non-scientific family and friends?**

The first steps in vision are performed by photoreceptor cells, which capture light and generate an electrical signal that gets transmitted to the brain. Many visual diseases that affect humans involve the degeneration of photoreceptors. In several animal models of these photoreceptor degenerative diseases, extracellular vesicles have been shown to accumulate in the space surrounding photoreceptors. However, the exact origin and the biological significance of these vesicles has been unclear. In this work, we show that healthy photoreceptors also release extracellular vesicles, although at a greatly reduced frequency compared to diseased cells. These vesicles contain a variety of cargoes, including mislocalized protein and mitochondrial material. Within the context of published work, our data suggest that the massive release of extracellular vesicles in diseased conditions represents an amplification of the normal, homeostatic process that is involved in the removal of mislocalized or damaged proteins from the cell.

“[…] the massive release of extracellular vesicles in diseased conditions represents an amplification of the normal, homeostatic process that is involved in the removal of mislocalized or damaged proteins from the cell.”

**What are the potential implications of these results for your field of research?**

This study opens up a lot of avenues for future research. For example, what is the overall impact of this vesicular release on maintaining the healthy status of normal photoreceptors? Is the massive level of vesicular release in photoreceptor degenerative conditions helpful or harmful? What are the molecular mechanisms underlying this release? What is the full scope of material that is in these vesicles? Ultimately, addressing these questions will allow us to better understand the significance of this phenomenon, which would be essential for identifying whether modulating vesicular release could serve as a potential therapeutic approach for treating a group of visual diseases.

**What are the main advantages and drawbacks of the experimental system you have used as it relates to the disease you are investigating?**

There are two types of photoreceptor cells: rods and cones. One big disadvantage of traditional research models, such as mice, is that they are rod dominant, with >95% of photoreceptors being rods. This severely limits the research that can be done with traditional models looking into cone photoreceptor structure and function. One very exciting part of this study was that we were able to study a cone-dominant species, the 13-lined ground squirrel, that has ∼95% of photoreceptors being cones! Taking advantage of non-traditional animal models, such as ground squirrels, will greatly help facilitate cone-specific research in the future.

**Figure:**
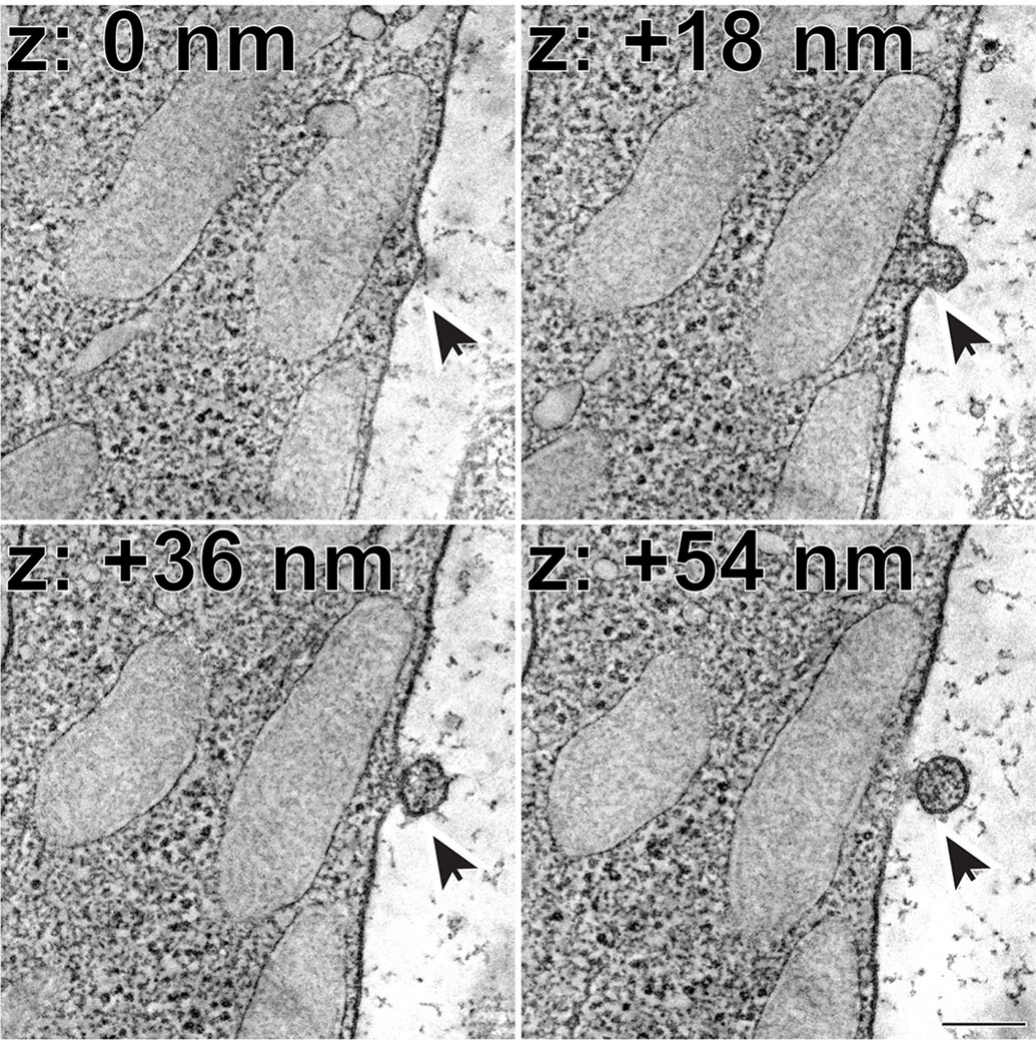
**Representative *z*-sections at the depths of 0, +18, +36 and +54 nm obtained from a 3D electron tomogram of a wild-type mouse retinal section.** The arrow depicts a microvesicle in the process of being released from the plasma membrane of a photoreceptor inner segment. Scale bar: 0.2 µm.

**What has surprised you the most while conducting your research?**

This study was spun from an unexpected observation made during our thorough analysis of the 3D structure of photoreceptor cells. I find it fascinating how research can take you in a variety of unexpected directions.

**Describe what you think is the most significant challenge impacting your research at this time and how will this be addressed over the next 10 years?**

This project was focused on performing in-depth ultrastructural analyses of photoreceptors. While traditional transmission electron microscopy (TEM) is a valuable technique that has been around for decades, there are some limitations to its use. Primarily, the *z*-resolution of TEM is limited by the thickness of plastic section (typically ∼50-70 nm). In this project, I was fortunate to work with the National Center for Microscopy and Imaging Research and employ a technique called electron tomography. Electron tomography involves taking a series of images over a tilt series, which provides a *z*-resolution on the order of a single nanometer, or ∼50-fold greater than that of TEM. The use of this method allowed us to visualize vesicles actively budding from the photoreceptor plasma membrane. I believe that employing modern techniques, such as electron tomography, will enable us to visualize previously unknown cellular structures and open up many new lines of research in the future.

“[…] openness for collaboration is to the benefit of all researchers […]”

**What changes do you think could improve the professional lives of scientists?**

I have been very fortunate over my research career to be involved in a wide variety of exciting and productive collaborations. I believe that an openness for collaboration is to the benefit of all researchers, and I am hoping that the good fortune that I have had in this regard continues in the future!

**What's next for you?**

After getting a K99 grant this year, I am planning on finishing a few projects as a postdoc before applying for faculty positions in the fall of 2023. I am looking forward to the job search process and seeing where I end up starting my own lab!
